# Self-reported depression and anxiety among COPD patients. A case-control study

**DOI:** 10.1590/1516-3180.2021.0235.R1.17062021

**Published:** 2022-02-21

**Authors:** Rafael Hurtado-Ruzza, Óscar Álvarez-Calderón-Iglesias, Ricardo Becerro-de-Bengoa-Vallejo, César Calvo-Lobo, Marta San-Antolín, Marta Elena Losa-Iglesias, Carlos Romero-Morales, Daniel López-López

**Affiliations:** I MD, PhD. Research, Health and Podiatry Group, Department of Health Sciences, Faculty of Nursing and Podiatry, Universidade da Coruña, Ferrol, Spain; and Otolaryngologist, Department of Otorhinolaryngology, Complexo Hospitalario de Ourense, Ourense, Spain.; II MD, PhD. Research, Health and Podiatry Group, Department of Health Sciences, Faculty of Nursing and Podiatry, Universidade da Coruña, Ferrol, Spain; and Otolaryngologist, Department of Otorhinolaryngology, Complexo Hospitalario de Ourense, Ourense, Spain.; III RN, BSc, MLIS, DPM, DHL, PhD. Full Professor, Department of Nursing, School of Nursing, Physiotherapy and Podiatry, Universidad Complutense de Madrid, Madrid, Spain.; IV PT, MSc, PhD. Assistant Professor, Department of Nursing, School of Nursing, Physiotherapy and Podiatry, Universidad Complutense de Madrid, Madrid, Spain.; V PSYCH, MSc, PhD. Assistant Professor, Department of Psychology, Universidad Europea de Madrid, Villaviciosa de Odón, Madrid, Spain.; VI RN, MSc, PhD, DPM. Full Professor, Department of Nursing and Stomatology, School of Health Sciences, Universidad Rey Juan Carlos, Alcorcón, Spain.; VII PT, MSc, PhD. Senior Lecturer, Department of Physiotherapy, Faculty of Sport Sciences, Universidad Europea de Madrid, Villaviciosa de Odón, Madrid, Spain.; VIII BSc, MSc, MPH, PhD, DPM. Senior Lecturer and Researcher, Health and Podiatry Group, Department of Health Sciences, School of Nursing and Podiatry, Universidade da Coruña, Ferrol, Spain.

**Keywords:** Depression, Anxiety, Pulmonary disease, chronic obstructive, Depressive symptoms, Anxiousness, COPD

## Abstract

**BACKGROUND:**

Anxiety and depression are the most prevalent mental disorders worldwide. However, the exact mechanisms linking chronic obstructive pulmonary disease (COPD) with depression and anxiety have not been identified.

**OBJECTIVES:**

To compare self-reported depression and anxiety among patients diagnosed with COPD in relation to healthy controls.

**DESIGN AND SETTING:**

Case control study at a public hospital institution in Spain.

**METHODS:**

We designed a case-control study. Patients were recruited using a consecutive sampling method from a single institution. Two groups were created: COPD and healthy controls. Data on medical history and demographic background were collected from the medical records. Self-reported depression levels were assessed using Beck’s depression inventory (BDI). Self-reported anxiety was measured using the State-trait anxiety inventory (STAI).

**RESULTS:**

Fifty-two patients with COPD and fifty healthy patients were included in this study. BDI scores were higher for COPD patients (10.23 ± 6.26) than in the control group (5.2 ± 6.56). STAI-state scores were higher for COPD patients (41.85 ± 12.55) than for controls (34.88 ± 9.25). STAI-trait scores were higher for COPD patients (41.42 ± 10.01) than for controls (34.62 ± 9.19).

**CONCLUSIONS:**

This study showed that there were higher levels of depression and anxiety among COPD patients than among healthy controls.

## INTRODUCTION

Chronic obstructive pulmonary disease (COPD) is currently the fourth leading cause of death worldwide.^1^ It is a major cause of chronic morbidity and mortality throughout the world. Many people die prematurely from it or its complications.^2^

COPD is defined as a respiratory disease, mainly caused by tobacco abuse and characterized by persistent symptoms such as chronic cough with or without expectoration and chronic airflow limitation, that usually manifests as progressive dyspnea.^3^

According to the World Health Organization (WHO), common mental disorders refer to two main diagnostic categories: depressive disorders and anxiety disorders. Both of these are highly prevalent in the population and can affect people of all ages. Over 300 million people are estimated to suffer from depression, equivalent to 4.4% of the world’s population, and this number seems to be increasing.^3^

Depressive disorders are characterized by symptoms such as sadness, loss of interest or pleasure, feelings of worthlessness or guilt, sleep difficulties, fatigue, appetite or weight changes, feelings of tiredness, psychomotor disturbances, poor ability to concentrate and even suicidality.^4^ Depression can be long-lasting or recurrent, and causes impairment to activities of daily life.

Anxiety is associated with physical and psychological discomfort. All anxiety disorders share common symptoms, such as fear, anxiety and avoidance. Other anxiety-related symptoms include fatigue, restlessness, irritability, sleep disturbances, reduced concentration, lack of memory and muscle tension.

Depression and anxiety often co-occur. Up to 90% of patients with anxiety develop symptoms of depression, and nearly 85% of patients with depression show some kind of anxiety symptom.^5,6^ The prevalence of depression and anxiety is two to three times higher among people with chronic medical conditions than among healthy people.^7^ People with a long-term condition and depression or anxiety have worse health status than people with depression or anxiety alone, and even people with any combination of long-term conditions without depression.^8^

Nearly 40% of the population are affected by an anxiety disorder at some point.^9^ The consequences of both anxiety and depression in terms of loss of health are substantial and have considerable effects on patients’ health-related quality of life. Depression is considered to be the largest single contributor to overall disability around the world.^10^ The most common underlying disorder among individuals who attempt suicide is depression.^11^

We hypothesized that people with COPD would have worse levels of self-perceived depression and anxiety traits and states, compared with healthy people.

## OBJECTIVE

The main objective of this study was to determine self-perceived depression and anxiety trait and state levels in a subset of COPD patients, compared with healthy people. To our knowledge, this was the first time that both Beck’s depression inventory (BDI) (second edition) and the state-trait anxiety inventory (STAI) had been used altogether to evaluate depression and anxiety among COPD patients, in comparison with healthy participants.

## METHODS

### Study design

The guidelines for reporting observational studies of the “Strengthening the Reporting of Observational Studies in Epidemiology” (STROBE) statement were followed.^12^ A case-control study was designed to compare self-assessed depression severity, anxiety traits and anxiety states among patients diagnosed with COPD and among healthy controls.

### Ethics statement

This study was approved by the ethics committee for clinic research of Galicia, Spain, under registration number 2019/431, with the application date of October 22, 2019. The Helsinki declaration and all national and international ethical standards for human experimentation were respected.^13^ Furthermore, all participants signed an informed consent statement before their inclusion in the present research.

### Sample size calculation

In order to determine the sample size required to ascertain differences between two different means, we applied a T test by using the G*Power 3.1.9.2 software (Heinrich-Heine-Universität Düsseldorf; Düsseldorf, Germany). We focused on BDI as our principal outcome measurement in the preliminary study (n = 28 participants) with two groups (mean + standard deviation, SD): 14 patients with COPD (9.28 ± 6.16 points) and 14 healthy controls (4.92 ± 3.64 points). Moreover, the following variables were used for the sample size calculation: effect size of 0.86, α error of 0.01 and power (1-β error) of 0.90.

In the end, the sample size determined through adjustments to calculations was 84 participants, i.e. 42 for each group, which was found necessary in order to achieve an actual power of 0.901. From this, a total sample of 150 subjects with 50 participants in each group was included in the present study, taking into consideration a possible 30% loss due to errors in data acquisition or incomplete questionnaires.

### Participants

We used a consecutive sampling method in order to recruit participants from the pneumology department of the Complexo Hospitalario de Ourense, Ourense, Spain. All the data collection was supervised by the same researcher. We established two groups: COPD (case group) and healthy participants (control group). COPD patients were diagnosed and classified in accordance with the GesEPOC guidelines (Spanish practical guidelines for COPD patient diagnosis and treatment).^14^ The inclusion criteria for both groups were that the subjects needed to be older than 18 years of age, agree to sign the informed consent statement and show a lack of history of psychiatric disease or use of antidepressants or anxiolytics. For the control group, healthy participants older than 18 years of age were included, without any chronic respiratory disease or known psychiatric medical history, and with agreement to sign the informed consent statement. The exclusion criterion was a lack of compliance with the inclusion criteria described above.

### Descriptive data

Descriptive data on sex, age, weight, height, body mass index (BMI; calculated through the Quetelet index as kg/m^2^)^15^ and smoking habit were collected.

### Outcome measurements

Self-perceived depression, anxiety traits and states were considered to be the primary outcomes.

#### Self-reported depression

We used the “BDI-II”, which is a widely used instrument for assessing the severity of depression worldwide. This tool has been validated for use in Spanish, with Cronbach’s α = 0.87 and high diagnostic validity (receiver operating characteristic, ROC = 0.91) in the general population.^16^

This questionnaire consists of 21 groups of sentences that are scored on a 4-point Likert scale (scores of 0-3). The results can range from 0 to 63 points. Greater scores suggest increased severity of depression. As an example, the first group of sentences states: *“I do not feel sad”* (0 points), *“I feel sad”* (1 point), *“I am sad all the time and I can’t snap out of it”* (2 points) and *“I am so sad and unhappy that I can’t stand it”* (3 points).

Total scores of 0-13 indicate minimal signs of depression; 14-19, mild depression; 20-28, moderate depression; and 29-63, severe depression.^17^

#### Self-reported anxiety trait and state

The STAI is a self-reported scale that is used for assessment of anxiety states (STAI-S) and anxiety traits (STAI-T) in research and clinical practice. This inventory consists of 40 statements about the participants’ feelings, divided into two parts. In the first part, they are instructed to indicate the intensity of their feelings of anxiety at a particular moment (state anxiety), on a 20-item scale. In the second part, they describe how they generally feel (trait anxiety) by reporting the frequency of their symptoms of anxiety, again on a 20-item scale. Both parts use 4-point Likert scale scores ranging from 1 (hardly ever) to 4 (often).^18^

The items in both STAI-S and STAI-T can score positively (for example, item 13 from the STAI-T *“I feel secure”*). However, there are also items that score negatively (for example, item 17 from the STAI-S *“I am worried”*). The final result is obtained using a correction factor. Higher scores suggest higher levels of anxiety. The Spanish version of the STAI was used and the final score was converted to a number on a scale from 0 to 80, as described by Buela-Casal, to be uniform with the original version of the STAI.^19,20^ This test has good internal consistency, with Cronbach’s α = 0.92 for anxiety states (95% confidence interval, CI: 0.91-0.93) and Cronbach’s α = 0.91 for anxiety traits (95% CI: 0.90-0.92).^21^ The test-retest reliability coefficients on initial development ranged from 0.31 to 0.86.^18^

### Statistical analysis

Statistical analyses were carried out by means the of the SPSS software, version 25.0 for Windows (IBM, Armonk, New York, United States), using an α error of 0.05 in conjunction with a 95% CI.

For quantitative data, the Kolmogorov-Smirnov test was applied to evaluate normality. All data were described as the mean ± SD and range (minimum-maximum), given that the median ± interquartile range did not accurately reflect the differences for some nonparametric data. For parametric data (Kolmogorov-Smirnov P-value ≥ 0.05), between-group differences were analyzed by means of Student t tests for independent samples. For nonparametric data (Kolmogorov-Smirnov P-value < 0.05), between-group differences were analyzed by means of Mann-Whitney U tests for independent samples. For categorical data, frequencies and percentages were applied to describe these values and their between-group differences were analyzed by means of Fisher exact tests and chi-square (χ^2^) tests.

## RESULTS

The study population included 102 participants: 60 men and 42 women. Their sociodemographic characteristics are shown in Table 1. Ten participants (9.80%) were smokers, 50 (49.01%) were former smokers and 42 (41.17%) were non-smokers. In the COPD group, there were 52 patients (10 females and 42 males), with a mean age of 70 ± 10.46 years. Only three patients were active smokers, while 45 were former smokers. The patients were treated with inhaled steroids, short-acting beta agonist (SABA), long-acting beta agonist (LABA), short-acting muscarinic antagonist (SAMA), long-acting muscarinic antagonist (LAMA) and alpha-1 antitrypsin. The control group was formed by 50 healthy participants (64% female and 36% male), with a mean age of 42.84 ± 15.69 years. None of them were receiving chronic medication.

**Table 1.** Sociodemographic and clinical characteristics of the sample population

	Sample	Control	COPD	P-value
	Mean ± SD (range) n = 102	Mean ± SD (range) n = 50	Mean ± SD (range) n = 52	
Age (years)	56.70 ± 19.4007 (19-88)	42.84 ± 15.69 (19-83)	70.02 ± 10.47 (36-88)	< **0.001** ^†^
Weight (kg)	72.59 ± 13.83 (43-115)	70.37 ± 13.9 (47-115)	77.06 ± 14.87 (47.5-120)	0.017^†^
Height (m)	1.68 ± 0.083 (1.50-1.89)	1.69 ± 0.92 (1.50-1.89)	1.68 ± 0.74 (1.50-1.83)	0.740^†^
BMI (kg/m^2^)	25.89 ± 3.98 (17.59-37.04)	24.48 ± 3.42 (17.59-33.79)	27.25 ± 4.04 (19.20-37.04)	< **0.001** ^†^
Sex (female/male)	42/60	32/18	10/42	< **0.001** ^‡^
Smoker (no/yes/former)	42/10/50	38/7/5	4/3/45	< 0.001**

COPD = chronic obstructive pulmonary disease; BMI = body mass index; SD = standard deviation; P < 0.05 with a 95% confidence interval was considered statistically significant. ^†^Mann-Whitney U test was used; ^‡^Fisher exact test was used; **Chi-square (χ^2^) test was used.

Fifty-two patients with COPD and fifty healthy patients were included in this study. The BDI scores were higher for COPD patients (10.23 ± 6.26) than the control group (5.2 ± 6.56). The STAI-state score (P < 0.005) was higher among the COPD patients (41.85 ± 12.55) than among the controls (34.88 ± 9.25). The STAI-trait scores (P < 0.001) were also higher among the COPD patients (41.42 ± 10.01) than among the controls (34.62 ± 9.19). These data are shown in Table 2.

**Table 2.** Depression and anxiety score differences between COPD and healthy participants

	Sample	Control	COPD	P-value
	Mean ± SD (range) n = 52	Mean ± SD (range) n = 50	Mean ± SD (range) n = 52	
STAI-State	38.43 ± 11.54 (20-68)	34.88 ± 9.25 (21-66)	41.85 ± 12.55 (20-68)	**0.005** ^†^
STAI-Trait	38.03 ± 10.20 (20-65)	34.62 ± 9.19 (20-65)	41.37 ± 10.10 (22-61)	< **0.001** ^†^
BDI	7.76 ± 6.86 (0-36)	5.20 ± 6.56 (0-36)	10.23 ± 6.26 (0-24)	< **0.001** ^†^

COPD = chronic obstructive pulmonary disease; SD = standard deviation; STAI = State-Trait Anxiety Inventory; BDI = Beck Depression Inventory; P < 0.05 with a 95% confidence interval was considered statistically significant. ^†^Mann-Whitney U test was used.

In the case group, we found that 36 patients had scores in the range 0-13, which indicated minimal signs of depression; ten patients were within the range 14-19, which indicated mild depression; six patients were within the range 20-28, which indicated moderate depression; and none were within the range 29-63, which would have indicated severe depression.^17^ In the control group, we found that 47 patients were within the range 0-13 (minimal signs of depression); one patient was within the range 14-19 (mild depression); one patient was within the range 20-28 (moderate depression); and one patient was within the range 29-63 (severe depression).

COPD could be classified into three subgroups, according to its severity, as shown in Table 3:

- Mild COPD: with a BDI score of 11.15 ± 6.78; STAI-S score of 44.15 ± 12.63; and STAI-T score of 42.92 ± 11.27.
- Moderate COPD: with a BDI score of 7.38 ± 5.12; STAI-S score of 38.31 ± 11.63; and STAI-T score of 38.63 ± 8.72.
- Severe COPD: with a BDI score of 11.70 ± 6.60; STAI-S score of 43 ± 12.21; and STAI-T score of 42.52 ± 10.14.

**Table 3.** Depression and anxiety score differences between COPD severity classification subgroups

	Mild COPD	Moderate COPD	Severe COPD	P-value
	Mean ± SD (range) n = 13	Mean ± SD (range) n = 16	Mean ± SD (range) n = 23	
BDI score	11.15 ± 6.78 (1-21)	7.38 ± 5.12 (0-21)	11.70 ± 6.60 (2-24)	< **0.001** ^†^
STAI-S	44.15 ± 12.63 (20-68)	38.31 ± 11.63 (20-60)	43 ± 12.21 (24-66)	< **0.001** ^†^
STAI-T	42.92 ± 11.27 (24-59)	38.63 ± 8.72 (26-54)	42.52 ± 10.14 (22-61)	< **0.001** ^†^

COPD = chronic obstructive pulmonary disease; SD = standard deviation; BDI = Beck Depression Inventory; STAI-S = State-Trait Anxiety Inventory - State; STAI-T = State-Trait Anxiety Inventory - Trait; P < 0.05 with a 95% confidence interval was considered statistically significant. ^†^Mann-Whitney U test was used.

## DISCUSSION

The relationship between COPD and depression is likely to be bidirectional, considering that depression may be both a consequence of COPD and also the cause of increased morbimortality. Depression or anxiety confers an increased risk of exacerbation of COPD and possibly death. Moreover, COPD increases the risk of developing depression.^22^ However, the exact mechanisms linking COPD with depression and anxiety have not been identified.

In the present study, we found higher self-reported anxiety state scores among COPD patients than among healthy participants. Bailey et al.^23^ described the so-called breathlessness/anxiety/breathlessness cycle and explained how, when people with COPD experience dyspnea, they become anxious. This anxiety causes more breathlessness, and thus these individuals enter a vicious circle of increasing anxiety and breathlessness. An increment in the patient’s experience of dyspnea will cause emotional distress and/or some form of help-seeking action. The most common help-seeking behavior often includes emergency admission to a hospital.^23^

The anxiety trait scores among patients with COPD were also higher than those of healthy participants. This can be explained through the understanding that patients with COPD know that their disease is irreversible and progressive. Lung disease limits the daily activities of many of these patients, and many of them are forced to change their occupation or even to retire prematurely. In addition, their social relations are also adversely affected.^24^ Associations between anxiety disorders and COPD appear to be largely explained by confounding factors, such as previous history of cigarette smoking and nicotine dependence.^25^

Regarding self-reported depression, the BDI scores among COPD patients in our study were higher than those of healthy participants. This was consistent with the data previously published by Schneider et al.^26^ and Jiménez-Cebrián.^27^ These authors reported that people with other chronic diseases, for example Parkinson disease, had results similar to those of patients with COPD, with higher prevalence of depression than in the general population. Depression itself is a risk factor for development of chronic illnesses such as diabetes and coronary heart disease, and it adversely affects the course and management of chronic medical illness.^7^ Moreover, the relative risk of developing depression is higher among patients with severe COPD.

Although the BDI, STAI-S and STAI-T scores were higher in the case group, we did not find any direct correlation between COPD severity and the scores of the three questionnaires. Goodwing et al. reported that the relationship between COPD and depressive disorders could be attributable to confounding due to cigarette smoking and lifetime nicotine dependence.^25^

There is also another possible explanation: the neurobiological theory. This maintains that depression is associated with activation of some aspects of cellular immunity, thus resulting in hypersecretion of proinflammatory cytokines and hyperactivity of the hypothalamic-pituitary-adrenal axis. Inflammatory deregulation as the etiological factor appears to be a plausible hypothesis for explaining why up to 50% of depressive patients do not respond to conventional therapy. The levels of pro-inflammatory cytokines in the brain can cause neurotransmitter imbalance, neuroinflammation and neurodegeneration.^28^

Recognizing the possibility of a psychological disorder in COPD patients is very important, in order to be able to establish adequate treatment if needed. Untreated anxiety and depression among patients with COPD have negative consequences for both patients and their caregivers, and may increase healthcare service utilization.^29^ Both anxiety and depression are associated with worse quality of life, greater functional deterioration and mortality, especially in the case of depression.^14^ The bidirectional association and the adverse prognostic impact of comorbid COPD and depression or anxiety need to be taken into consideration by physicians.^22^ Some good results have been obtained through treatments consisting of pulmonary rehabilitation, smoking cessation and psychological and antidepressant drug therapy, for reducing anxiety and depressive symptoms among patients with COPD.^29^

### Future studies and clinical implications

#### Limitations

Some limitations of our study need to be mentioned. A consecutive sampling method was used in order to recruit participants and should be considered in future studies. Although this was significant, a larger matched-paired sample size might be necessary to achieve more reliable results. Also, a further study taking the role of medication into consideration should be conducted.

The use of self-reported questionnaires, even though they have been validated, does not replace diagnoses of specific anxiety or depressive disorders that are made by a qualified mental health professional through a structured clinical interview.

## CONCLUSIONS

COPD patients showed higher scores for self-reported depression, anxiety state and anxiety trait than controls.
